# Selection against Accumulating Mutations in Niche-Preference Genes Can Drive Speciation

**DOI:** 10.1371/journal.pone.0029487

**Published:** 2011-12-27

**Authors:** Niclas Norrström, Wayne M. Getz, Noél M. A. Holmgren

**Affiliations:** 1 Systems Biology Research Centre, University of Skövde, Skövde, Sweden; 2 Department of Environmental Sciences, Policy and Management, University of California, Berkeley, California, United States of America; 3 School of Mathematical Sciences, University of KwaZulu-Natal, Durban, South Africa; University of California Santa Barbara, United States of America

## Abstract

Our current understanding of sympatric speciation is that it occurs primarily through disruptive selection on ecological genes driven by competition, followed by reproductive isolation through reinforcement-like selection against inferior intermediates/heterozygotes. Our evolutionary model of selection on resource recognition and preference traits suggests a new mechanism for sympatric speciation. We find speciation can occur in three phases. First a polymorphism of functionally different phenotypes is established through evolution of specialization. On the gene level, regulatory functions have evolved in which some alleles are conditionally switched off (i.e. are silent). These alleles accumulate harmful mutations that potentially may be expressed in offspring through recombination. Second mating associated with resource preference invades because harmful mutations in parents are not expressed in the offspring when mating assortatively, thereby dividing the population into two pre-zygotically isolated resource-specialist lineages. Third, silent alleles that evolved in phase one now accumulate deleterious mutations over the following generations in a Bateson-Dobzhansky-Muller fashion, establishing a post-zygotic barrier to hybridization.

## Introduction

The relevance of sympatric speciation, as opposed to allopatric speciation, in nature has been controversial. It has been a theoretical issue to understand how evolutionary bifurcation can occur when disruptive selection is opposed by inter-breeding in the population. Focusing on the dichotomy between allopatric and sympatric speciation is questionable; rather there is a plea for research on the speciation processes and its mechanisms [1]. Genetic studies of sympatric populations exhibiting a monophyletic origin suggest recent ecological divergence, reproductive isolation, and speciation without geographical barriers [2]–[5]. Particularly striking is the colonization of post-glacially emerging habitats by marine snails and sticklebacks, in which bifurcation has occurred repeatedly across sites and in parallel giving rise to homologous phenotypes [6]–[9]. These studies raise the issue of mechanisms behind (i) evolutionary diversification and (ii) the maintenance of apparent lineages: mechanisms that need not be the same for both processes [10]. Sympatric speciation is currently understood in terms of two consecutive processes. First, loci for niche-specific adaptations give rise to multiple alleles in a multi-niche environment. Second, assortative mating evolves to reduce heterozygotes that are poorly adapted. These processes have been analyzed using 1–2 loci models with discrete alleles [11]–[15], and with continuous alleles [16]. In a model of Dieckmann & Doebeli [17], quantitative traits are under the control of multiple loci interacting additively. Furthermore, they assume that the niche-breadth of the individuals is fixed in a continuum of resources. Such models, for example, apply to Darwin's finches in the Galapagos Islands, where disruptive selection acts on mouthpart morphology in the context of scramble competition in an environment with a range of variably sized prey. Under these circumstances, the assumption of additive genetics implies that morphologically intermediate heterozygotes have reduced fitness induced by intense competition [17]. This competition promotes selection for assortative mating that will evolve if ecological genes (genes for adaptation to a niche) have a pleiotropic effect on mating [15], or a mating preference gene that is in linkage disequilibrium with the ecological gene for which it expresses preference [18], [19]. In the first case, the existence of such genes (called magic genes) may seem obscure [20], but it is not uncommon that mating takes place in locations where preferred resources abound. The required linkage in the second case, however, imposes a restriction on the likelihood of sympatric speciation [21]. However, evolutionary branching of a genetic lineage is one plausible outcome of disruptive selection against intermediate phenotypes while evolution of dominance and a protected polymorphism is another [22], [23]. When trait-specific dominance has evolved, there is no disadvantage for heterozygotes and the selection for assortative mating and speciation has been exhausted [24].

In this paper we show that evolved polymorphism does not necessarily prevent selection for assortative mating. Instead assortative mating evolves due to costs of deleterious mutations on epistatic alleles essential to the polymorphism. We will also show that once assortative mating has evolved, the epistatic alleles are inactive and are a target for the evolution of Bateson-Dobzhansky-Muller incompatibility [25], [26]. In our case the precise mechanism relates to the following: 1.) Resources sometimes require resource-specific discrimination and cannot perceptually be generalized with other resources; 2.) A population of exploiters can utilize two such resources in a protected polymorphism of both homo- and heterozygote specialists; 3.) The haplotypes of the polymorphism carries two types of alleles: discrimation alleles and modifiers. In homozygotes discrimation alleles express resource-specific discrimation whereas modifers are silent. Alleles on heterozygotic loci will interact: modifier alleles alter or turn off the discrimator allele, or two different discrimator alleles interact codominantly altering or silencing the gene expression; 4.) Following from the genetic structure, some of the alleles are inactive, either in the homozygote or the heterozygote form. These alleles are susceptible to and can accumulate harmful mutations that are not subjected to selection until they are re-organized and expressed in a subsequent generation of offspring. 5.) Accumulated harmful mutations impose a cost for dis-assortative mating and hence selection for assortative mating occurs.

In highly specialized herbivores, parasites, and parasitoids, disruptive selection can operate on the niche-recognition trait itself. Striking examples are “cryptic species”, a pair (or guild) of species that are morphologically indistinguishable but select different species-specific hosts [27]. Correlations among haplotype sequences and host preference have led to previously regarded host-races of generalist species being assigned the status of “cryptic” species [28], [29]. Habitat preference has been studied with additive multilocus models, also including host-adaptation genes with opposite alleles being adapted to different hosts, called “Bush-models” [30]–[32]. Speciation has then been driven by evolving linkage between host-adaptation alleles and host-preference alleles, the latter also determining mating [32]. Here we model such niche (or host) preferences of exploiters using genetically-coded artificial neural nets (ANN) and, as in Bush models, without any niche adaptation genes under diversifying selection. ANNs have been used as models of neural and perceptual systems [33], [34] that are capable of non-linear discrimination of signals [35]. Individual nodes within ANNs participate in linear discrimination: in our model we identify such nodes that are controlled by epistatic genes. The nodal weightings of our ANNs are identified with a pair of chromosomes, subject to mutations. Our model contains additional elements that are identified as second chromosomal pair that holds a mating preference gene with a modifier allele for assortative mating, i.e. mating takes place on preferred resources [17], [36], a condition known as heteropatry [15]. A population of exploiters compete for resources in two niches identifiable by their ANN. The ANN enables the exploiters to evolve preference of any niche-breadth or modality (e.g. bimodality) without any pre-defined costs or trade-offs. These ANN automatons reproduce sexually, including chromosome recombination and crossover. In the simulated evolutionary process we study the emergence of reproductively isolated phenotypes with regard to their niche and mating preferences. In order to understand the selection at the level of emergent alleles, we dissect the evolutionary process into phases of distinct genetic organisation and selection pressure.

## Methods

### The Model

#### The environment

**Figure 1:**
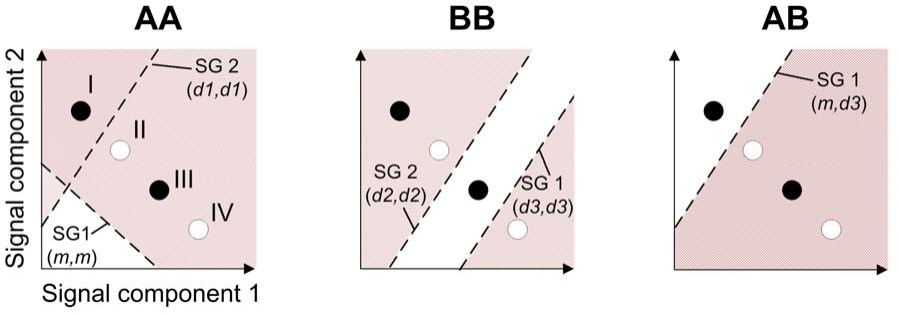
Evolved discrimination of four niches (I–IV) in signal space for homozygotes AA, BB and the heterozygote AB. Solid black dots represent beneficial niches and white dots detrimental niches, shown in the two-dimensional signal space. Dashed lines represent schematic linear discrimination by the hidden neurons 1 and 2 associated with the two super-genes SG1 and SG2. Genotype AA is homozygote in SG2 with *d_1_* that performs discrimination between resource I and II–IV, whereas the modifier *m* in SG1 does not perform any discrimination between resources in homozygote form. Genotype BB is homozygote with two discriminators, *d_2_* that discriminate resource I and II from III and IV, and *d_3_* that discrimate resource IV from the others. In SG1 of the heterozygote AB, m modifies the expression of *d_3_* to discriminate resource I from the others. SG2 of AB is heterozygote *d_1_d_2_* and does not perform any discrimination. The areas with stripes from upper-left to lower-right represent areas where the output neuron is inhibited whereas areas with stripes from lower-left to upper-right represent areas where the output neuron is excited.

We modelled a dynamic population of about 400 evolvable exploiters in an environment of two suitable and two unsuitable niches represented by the set of resource values [*N*_1_, *N*_2_, *N*_3_, *N*_4_]. We used the values [250, 0.01, 250, 0.01] in all simulations, where 250 and 0.01 individuals are the “carrying capacities” respectively of the suitable and unsuitable niches (the latter value is slightly above 0 to avoid division by 0 in computations). Unsuitable niches can be thought of as containing resources that are defended (e.g. chemically or physically) against consumers. The resources (*k* = 1,…,4) are assumed to be perceived by consumers through a two-channel signal set (*s_k_*_1_, *s_k_*_2_), where 0≤*s_kl_*≤1 for all *k* and *l*. In the context of plant-herbivore interactions, for example, the two components might be odorants of an odor signal, where the ratio of components and their total intensities are the salient cues. The signals in our simulations were [(0.2, 0.8), (0.4, 0.6), (0.6, 0.4), (0.8, 0.2)], which lines up the resources along a diagonal in the signal space (Fig. 1). This particular arrangement of the resources provides the most difficult discrimination task for the ANN, given the number of resources and dimensions of the signal space [37].

#### Exploiter perception and the ANN

**Figure 2:**
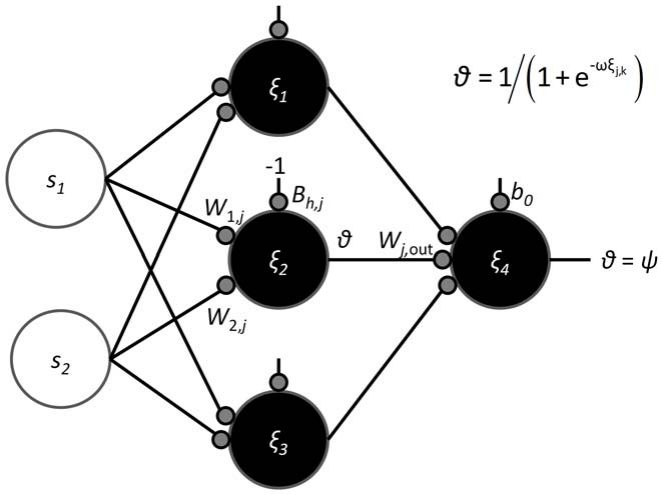
The architecture of the ANNs used in the simulations. Sensory nodes (open) simply propagate the signals elicited by the resource. Each hidden node *j*, *j* = 1,2,3 in the second layer has three ‘synaptic’ weights associated with it: *w*_1*j*_ and *w*_2*j*_ weight the inputs from sensory nodes 1 and 2 respectively and *w_j_*_ out_ weights the value of the output entering the third layer output node (shown for the middle node only). Further, the hidden nodes have bias weights *b_hj_*, and the output node the bias weight *b_o_* connecting an input of −1. Thus, each perceptron has a representation [**w**_1_, **w**_2_, **w**_3_, **w**_o_],where **w** *_j_* = (*w*_1*j*_, *w*_2*j*_, *b_h_*_j_, *w_j_*_ out_) for *j* = 1,2,3, are the hidden node values and **w**_o_ = *b*_o_ is the output node value (seen as colors in Fig. 3). The output *θ* that determines the response of each node is given by the sigmoidal threshold function (upper right corner) where, for sufficiently large *ω* (here = 4), an internal activity *ξ_jk_*<0 produces output close to 0, otherwise a value close to 1. For a hidden node *j* = 1,2,3 with niche (host) *k*'s signal applied, the activity values are determined by *ξ_jk_* = (*s_k_*_1_ *w*_1,*j*_+*s_k_*_2_ *w*_2,*j*_−*b_hj_*) and for the output neuron by *ξ*_4*k*_ = (*θ*_1*k*_ *w*_1,*out*_+*θ*_2*k*_ *w*_2,*out*_+*θ*_3*k*_ *w*_3,*out*_−*b*_0_). The output of the perceptron (*ψ*) is hence between 0 and 1 and interpreted as ‘approximately 0⇒avoid niche’ and ‘approximately 1⇒exploit niche’.

The signals are ‘perceived’ by exploiter perceptrons: a feed-forward ANN (Fig. 2) capable of non-linear discrimination if the number of layers are at least three. ANNs are models of biological neural circuits with nodes having the functionality of a neural cell [35]. We have chosen a three-layer perceptron architecture with two sensory input nodes (one for each signal channel), three hidden nodes (the minimum required to discriminate four resources), and one output node (Fig. 2). Sensory nodes propagate the signals to the hidden nodes through a weight (mimicking synapses of real neurons). The weighted signals excite or inhibit the node, which will switch its output (from 0 to 1 or the reverse) should the node excitation pass a threshold (the switch, being defined by a sigmoidal function as is explained below, effectively occurs over an interval rather than at a point). The slope of the threshold over the switching interval is controlled by a “bias-weight,” which is commonly used in ANNs. Depending on weight settings, the node switches around a certain ratio of the two input signals (Fig. 1). The output signals of the hidden nodes are similarly propagated via weights to the single output node. Depending on the value of the weights, the output node integrates the sigmoidal responses of the hidden nodes into an effectively off-on (0–1) response. The output from the ANN is interpreted as strength-of-preference for the niche signals (Fig. 1), which is 0 (avoid niche) and 1 (utilize niche).

#### The genetic model

**Figure 3:**
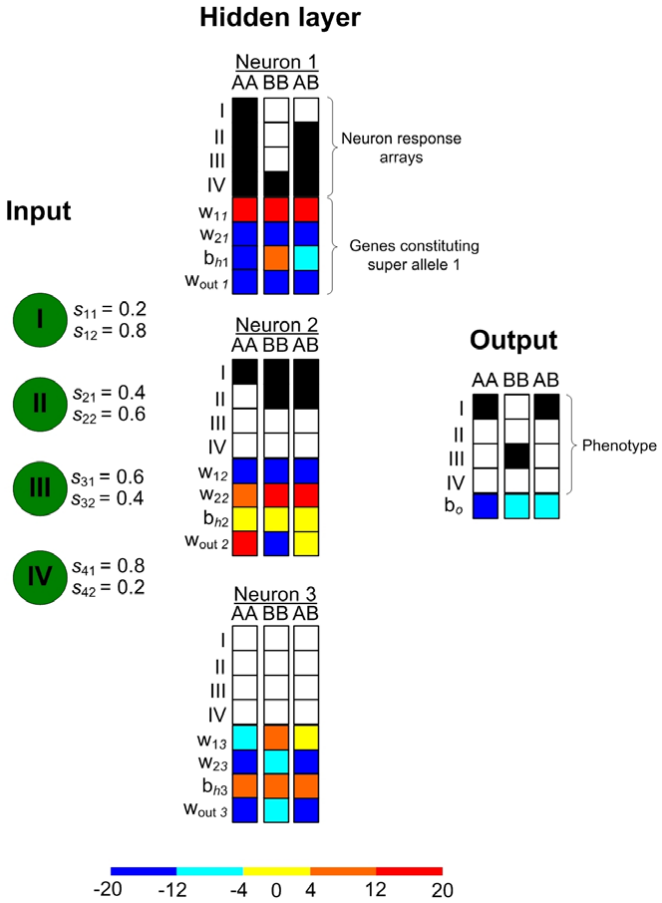
Weights (colour squares) and neural activity (b&w squares) to resources I–IV of the genotypes in the polymorphism. Niche signal components (*s_j_*_1_ and *s_j_*_2_, for resource *j* = 1≡I,2≡II,3≡III,4≡IV) propagate via weights (*w*_1*k*_, *w*_2*k*_) to the hidden neurons (*k* = 1,2,3). SG1 and SG2 code for these weights, the bias weight (*b_hk_*), and the output weight (*w*_out *k*_), which settings are represented by colours (bar at the bottom). The weights (row 5–8) and corresponding response (row 1–4; black: response >0.5; white: response <0.5) are given for genotypes (AA, AB, BB) and niches I–IV. For the output neuron, the fifth row represents the bias weight *b_o_*.

The 13 weights (parameters) in our perceptron automatons metaphorically represent 13 genes on a chromosome-pair. The alleles associated with these genes are assumed to be codominant: i.e. the weight of the synapse they are associated with is calculated by taking the average of the values of the two alleles. There are three sets of four genes each (*w*_1*j*_, *w*_2*j*_, *b_h_*_j_, *w*_out *j*_) that determine the contribution of the *j* ^th^ hidden neuron to the overall perceptron response to each of the resource signals. We interpret these four genes that are functionally associated with each node as ‘super-gene’ (SG) and its associated alleles a ‘super-allele’ (SA). This designation turns out to be useful for interpreting the evolutionary process. The order of genes on a chromosomal haplotype is *w*_11_, *w*_21_, *b_h_*_1_, *w*_12_, *w*_22_, *b_h_*_2_, *w*_13_, *w*_23_, *b_h_*_3_, *w*_out 1_, *w*_out 2,_ *w*_out 3,_ *b_o_* and linkage applies in our rules for combining haplotypes (cf. Fig. 3). An additional mating gene, unlinked to the genes on the perceptron chromosome, determines whether the exploiter mates on the resource of its preference (effectively a form of assortative mating) or randomly across all niches [36]. These associated mating alleles are designated *a* (assortative) and *r* (random) respectively, with allele *a* dominant over *r*. We have made runs with the opposite dominance, without any significant differences in the results. Our model elaborates on the details of the genes for niche preference, whereas the genetic coding for mating behaviour is highly idealized to facilitate assortative mating (and speciation) after niche preference has emerged (i.e. evolved).

#### The algorithm – exploiter fitness

Each exploiter senses each resource-niche through the application of the resource's signal to the exploiter's two sensory (input) nodes. The signals are propagated through each exploiter *i*'s perceptron one resource at the time, creating an output array Ψ = [*ψ_1,i_*, *ψ_2,i_*, *ψ_3,i_*, *ψ_4,i_*] for the response of this individual exploiter to niches I–IV, where resources *k* = 1…4 have the identification 1 = I, 2 = II, 3 = III, 4 = IV. The output response array is now the basis of the exploiter's (*i*) reproductive output (*e_k,i_*) prior to density-dependent effects on a resource *k*, which is computed using the formula
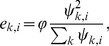
(1)where *ϕ* ( = 3) is the maximum reproductive output before progeny utilize any resources. In effect, the reproductive output depends on both the relative response (of resource *k* to the sum of all resources) and the absolute response, hence the squared *ψ*. If the reproductive rates are proportional to *ψ* rather than its square, simulations indicate that the ANN responses drift downwards towards zero. Hence eqn 1 has the effect that intermediate responses result in a reduced reproductive output, which implies selection for a bimodal (*ψ* = 0 or 1) [38] rather than gradual response to a niche landscape mapped onto the sensory input space. This has also the consequence that the reproductive output is the same for all exploiters expressing any of the 16 binary output arrays, regardless of being a specialist on one resource or generalist on all of them. Other models having a continuum of resources that usually apply convex niche-functions of fixed width [17]. In our model the ANN is capable of evolving niche-functions with multiple peaks of any width.

Next our model computes the fitness of exploiter *i* that includes a niche-specific density-dependent effect using the competition function
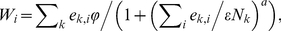
(2)where parameter *ε* ( = 1.5) sets the half-saturation density of the total reproductive output (Σ*_i_ e_k,i_*) in proportion to the carrying capacity (*N_k_*) of the competitive function and *a* ( = 2.5) determines the abruptness in the onset of density-dependence around the population density level *ε* [39]. This phenomenological function with a sigmoidal shape provides gradual selection potentials at extreme ends, and rapid evolution during transition phases. Thus our model incorporates two costs: the cost of using unfavorable resources and the cost of competition with other exploiters. No other explicit costs relating to the degree of resource specialization or assortative mating are included in the model. This does not mean that we regard such costs as uncommon in nature; the evolution of phenotype-genotype interactions is more transparent without them, and they can easily be included in elaborated studies.

#### The algorithm – mating

The exploiters are assigned to one of five mating pools: a random mating pool *p*_0_ and *p*_1_–*p*_4_ associated with the four niches. Mating genotypes *rr* belonging to pool *p*_0_ and select mates within the whole population, whereas genotypes *ra* and *aa* in the various output response phenotypes select mates in one of *p*_1_–*p*_4_ with probabilities in proportion to their their response array Ψ. Specifically, to categorize the exploiters' mating types, we used the rounded integer response values of Ψ. Thus niche 1 specialists [1000] were assigned to pool *p*_1_ with probability 1, selective-generalists [1010] were assigned to pools *p*_1_ and *p*_3_ with probabilities 0.5 respectively, and so on. The first exploiter of a pair was chosen at random from the whole population. It was paired with a random partner within its pools if it belonged to any of pools *p*_1_–*p*_4_ or with a random partner in the whole population if it belonged to pool *p*_0_. This has the consequence that individuals mating assortatively cannot choose to mate with individuals mating randomly. Paired exploiters were removed from the pool once mated, until there was either a single individual with no mate or no one was left. The number of offspring assigned to each reproducing pair equalled the nearest integer of the average fitness value of the individuals in the pair (eq. 2).

#### The algorithm – formation of recruits

The genotypes of the diploid offspring are created through sexual recombination of parental genes. At reproduction each exploiter produces a haploid gamete consisting of a preference chromosome and a mating chromosome. The preference chromosome is created by copying the alleles from one randomly chosen parental chromosome to the gamete, with a probability ( = 0.0001 per allele) of a cross-over at any locus. With the numbers in our simulation this amounted to an average of about one chromosome in the population exhibiting cross-over in each generation. We also allowed for point mutations to occur with probability 0.01 per allele. where the size of the change to the value of the weight mutation was drawn from a uniform probability distribution on (−10… 10), with the constraint to weight values to lie within the range (−20… 20). This avoided the weights drifting into very large or small (large negative) numbers. With this mutation rate, around 100 chromosomes on average were experienced to at least one point mutation each generation, but some changes were small and others constrained. Mating chromosomes were copied by randomly selecting one of the parental chromosomes, and when allowed were subject to a mutational event that transformed them from *r* to *a*, or the reverse, with probability 0.0005 (i.e. c. 0.4 mating chromosomes were altered in this way every generation). Model sensitivity to mutation rates and range of perturbations of the resource preference genes has been explored in a previous study [38]. Although higher mutation rates generally promote faster evolution and shorter simulation times, that study shows that increasing the rate of mutation in the preference genes from 0.01, as reported bere, to 0.15, made it much more unlikely for guilds that were matching the resources to evolve. Additionally, that study shows that the range of mutational perturbations of resource alleles has to be suffiently large (−3…3 was compared with −0.2…0.2) to enable simulations to escape local minima to find globally close-to-optimal solutions. Since the mutations on the mating genes have a strong and direct effect, we selected rates that provide a balance between the total mutational rates of the mating gene and the whole resource preference gene complex. The code is available on request.

#### The algorithm – iterations

The simulations were initialized with 500 individuals in the exploiter population. Each was assigned a diploid genome (two haploid neural chromosomes) with values at each of the 13 haploid loci drawn from a uniform probability distribution on (−1…1). This created a population of non-discriminating phenotypes with high preference for all habitats, and with some genetic variation (a standard initialization of ANNs) [35]. The evolutionary process was iterated over 100,000 generations where random mating was enforced during the first 20,000 generations by not allowing mutations to occur to the random mating allele initially assigned to all individuals. This constraint allowed time for a guild of exploiters of niches I–IV to evolve before continuing with the simulation to evaluate the process whereby mating alleles evolve and separate the population into assertive mating groups. Such groups then have the potential to genetically diverge and hence speciate. After 20,000 generations the random mating alleles were allowed to mutate to assortative mating, as well as back again to random. After 80,000 generations, we switched off the mutations to get rid off phenotypes continuously generated by mutations. This permits us to get beyond the initial conditions and study the evolutionary process of the emergence of fittest niche-preference alleles over the first 20,000 generation, evaluate the additional complexities of the action of habitat-linked mating genes over the next 60 thousand generations, and finally confirm the genotypes formed by selection alone (without mutations) over the final 20,000 generations.

### Analysis of genetic data

**Figure 4.Principal:**
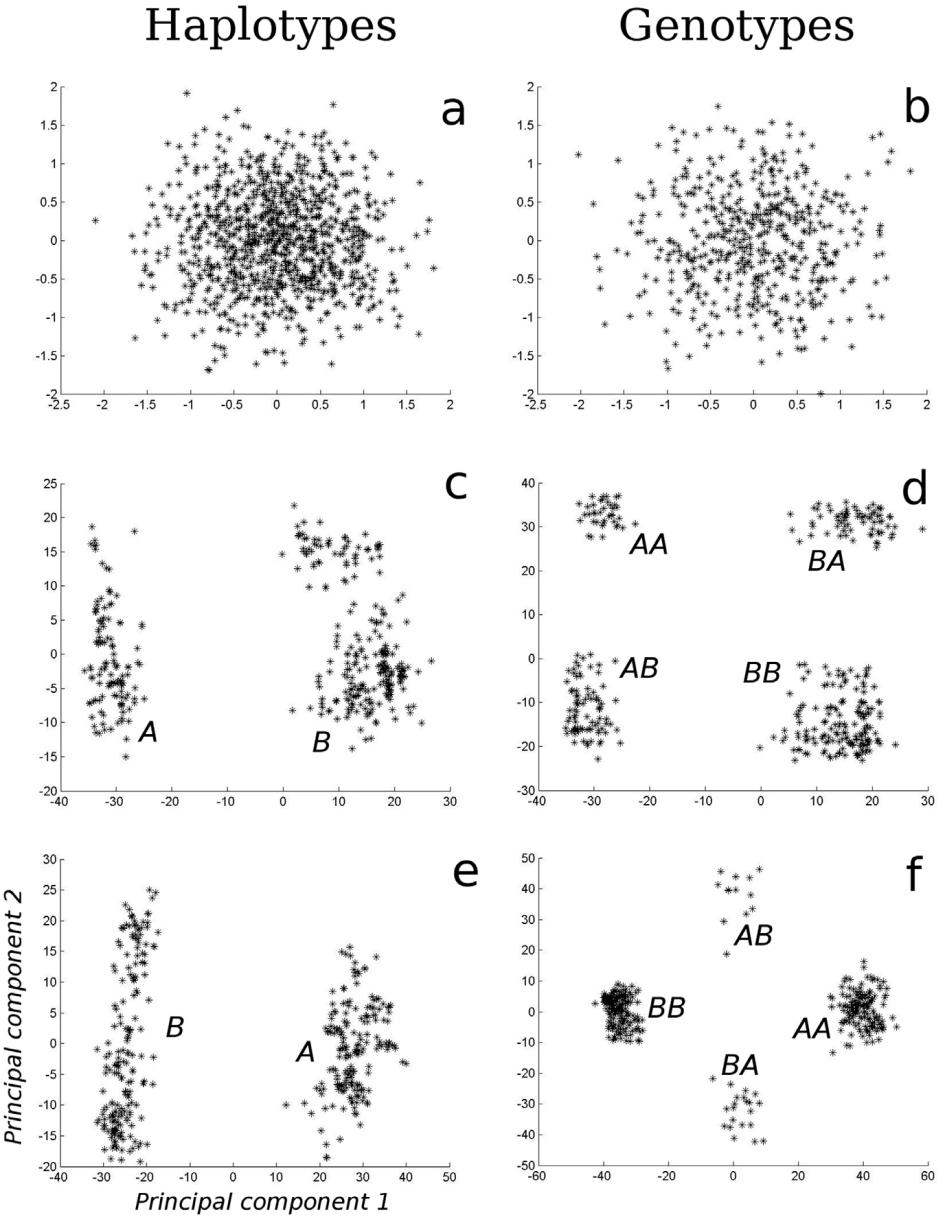
component analysis (PCA) on haplotypes (left column) and genotypes (right column) at different stages of the simulation. The initial condition is an even scatter of haplotypes (**a**). and genotypes (**b**). At generation 20,000 two haplotypes (A, B) and three genotypes (AA, AB, BB; AB and BA are separated only for reasons of how we represent genotype information) has evolved under random mating (**c** and **d**). The genotypes at generation 30,000 prevail after invasion of assortative mating (**e** and **f**,), with heterozygotes now more rare. Principal components are unique to each panel and hence cannot be compared across panels. Data is taken from the same simulation as in Fig. 6.

We used principle components analysis (PCA) to verify the existence of haplotypes (Fig. 4). Specifically, we used a Matlab® *princomp* algorithm to identify clusters and then plotted the haplotypes visually demonstrating that clustering had occured. Evolving phenotypes were classified to the nearest integer response array Ψ. Hence 16 phenotype classes are possible using such binary array representation. Thus, for example, [0,0,1,0] phenotypes are specialists phenotype on niche III while [1,1,1,1] are the non-discriminating generalists.

### Test of mutation robustness in phenotypes

Since evolution can be driven by selection for genetic robustness [40], [41] we sampled our simulation for exploiter individuals every 1000 generations on the interval 15,000–20,000 when the population was still under enforced random mating, and on the interval 27,000–32,000 to when assortative mating had evolved. After sorting individuals obtained from the above samples into genotypes, the proportion of phenotypes that were unfit was then evaluated, where unfit phenotypes are defined as those that show a preference for one or more unfavourable niches (with detrimental or non-viable resources). To assess the effects of mutations, *K* mutants were generated by randomly picking with replacement (i.e. returning sampled individuals to the pool unaltered) an individual from the phenotype group of interest and subjected it to a mutation (a weight perturbation as in the simulation) at a random locus. The sample size *K* = *P*(*P*−1), where *P* is the size of the phenotype pool, means that many individuals were resampled but the mutations are likely to differ. We then evaluated the number of unfit phenotypes among the *K* so produced. The percentage was multiplied by *P* and rounded off to the nearest integer to represent the expected number of unfit individuals in a genotype after being subjected to mutations. The increase in unfit individuals is expressed as a percentage of the original number.

## Results

### Evolving gene complex for resource selection

The initial 500 perceptrons at the start of each new simulation were assigned genetic values (i.e. node weightings and biases) at random, which accounts for the initial lack of clustering of haplotypes (Fig. 4a) and genotypes (Fig. 4b). Twenty thousand generations later, under random mating, two haplotypes (Fig. 4c) have emerged, creating three diploid genotypes in four clusters (Fig. 4d; clusters AB and BA represent the same genotypes but are clustered differently because the allele vectors are aligned in two ways). In the depicted case, haplotypes A and B constitute a stable polymorphism in which the homozygote genotype AA and the heterozygote genotype AB (Fig. 4d) express phenotype P1, a specialist on resource 1 (i.e. exploits niche I only) (Fig. 1), whereas homozygote genotype BB expresses phenotype P2, a specialist on resource 3 (i.e. exploits niche III only).

**Figure 5:**
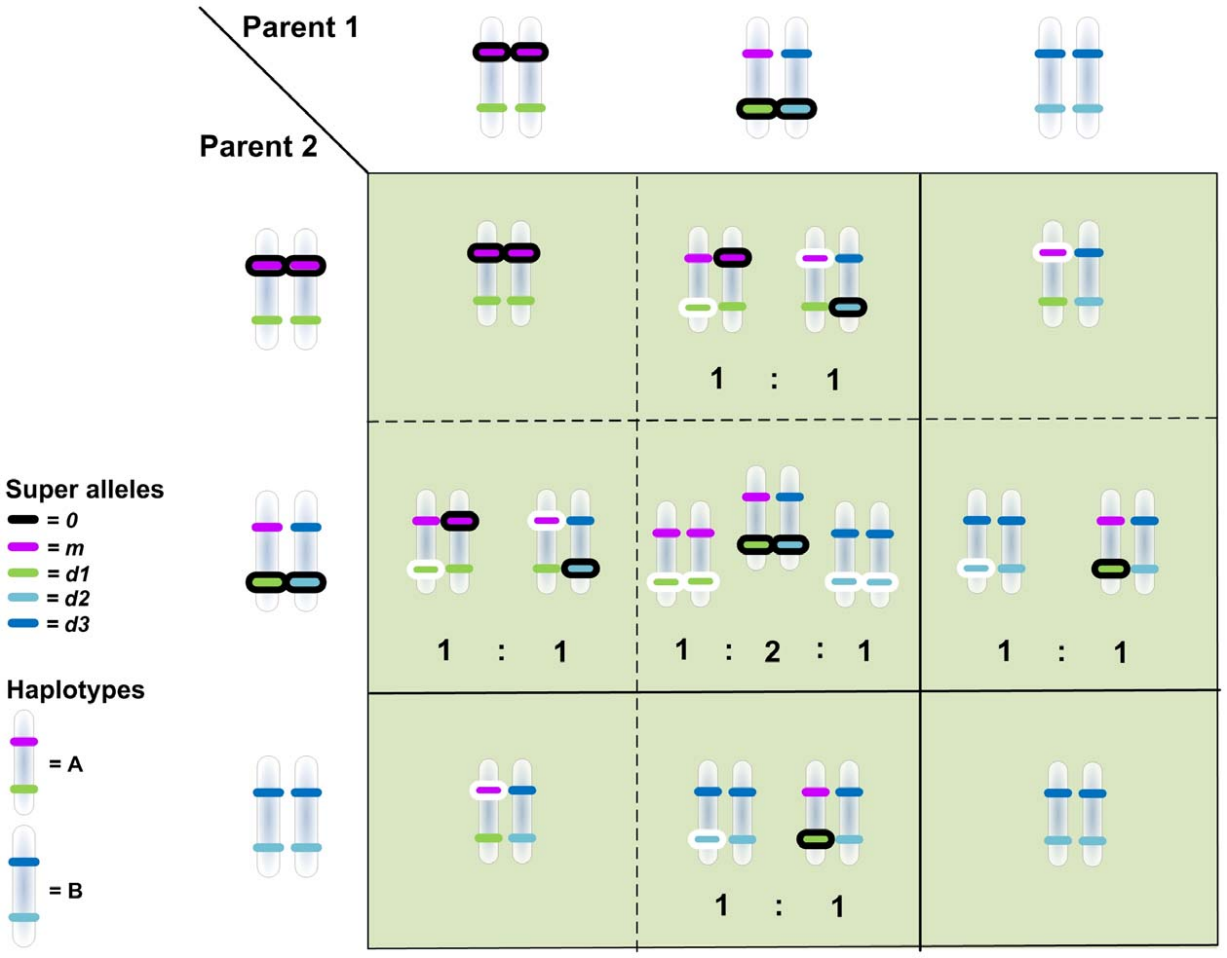
Inheritance and expression of harmful mutations in zygotic offspring. The Punnett square shows the reconfiguration of the haplotypes in the offspring of random mating individuals in the polymorphism. SAs, and haplotypes, are labelled according to the legend in the lower left corner of the figure. Silenced SAs, more likely than expressed SAs to accumulate harmful mutations, are indicated by black ovals encircling the allele. By recombination, the altered SAs are expressed in the offspring (indicated by a white oval) reducing its fitness. Homozygotes mating strictly assortatively are the only parents avoiding expression of harmful mutations in their offspring, thus there is selection for assortative mating. For clarity the figure illustrates only the inheritance of accumulated harmful mutations, without chromosomal cross-over that occurs rarely in the model. The proportional output of genotypes from mating is indicated as a ratio (e.g. 1∶2∶1). Solid lines represent the reproductive barrier induced by assortative mating where the four upper-left squares makes up the Punnett square from matings in niche 1, and the lower-right square shows the result from matings in niche 2.

During the simulation, four SAs (super-alleles, see *Methods, the genetic model*) *d*_1_, *d*_2_, *d*_3_ and *m* emerged in association with the SGs (super genes), arranged as haplotype A = *md*_1_0 and B = *d*_3_ *d*_2_0, where 0 is a marker for alleles not involved with discrimination. These give rise to the three genotypes AA = *mm d*_1_ *d*_1_ 00, BB = *d*_3_ *d*_3_ *d*_2_ *d*_2_ 00, and AB = *md*_3_ *d*_1_ *d*_2_ 00. In homozygous form the SAs *d*_1_, *d*_2_, and *d*_3_ respectively facilitate discrimination of niche I from the others, niches III and IV from the others, and niches I–III from IV. In homozygous form, the SA *m* does not facilitate discrimination (Fig. 1); but in heterozygous form SA *m* with SA *d*_3_ (genotype AB) modifies the hidden neuron response to favour niches I over II–IV (as a homozygote SA *d*_1_ would do). SAs *d*_1_ and *d*_2_ are mutually neutralized when together in heterozygote form (genotype AB) because *d*_1_ excites while *d*_2_ inhibits the output neuron (neuron 2, *w_out_*_ 2_, Fig. 3). Thus, there are SAs that can be functionally ‘silenced’, meaning that they are not involved in any discrimination. In the polymorphism with random mating, the consequence of ‘silencing’ is that mutations are not expressed in either SG1 of the AA genotype or in SG2 of the AB genotype. Mutations can thus accumulate each generation until the mutated allele becomes expressed by rearrangement in F1 or later generations: only then is the defective allele removed by selection (Fig. 5).

Out of ten repeated independent simulations checked at generation 20,000, six developed the type of polymorphism just described (the heterozygote and one of the homozygotes code for the same phenotype). The other four either developed a polymorphism with the two homozygotes expressing P1 and the heterozygote P2 (or the reverse; two simulations), or developed a monomorphic population of a discriminant generalists (phenotype [1,0,1,0]; two simulations). All of the six type cases of polymorphism evolved into homozygote specialists. In this presentation we focus on these type-cases to get deeper insights to the underlying mechanisms. The reader interested in the evolution of generalists and specialists is referred to our previous studies on asexual populations [37].

### Evolving assortative mating

**Figure 6:**
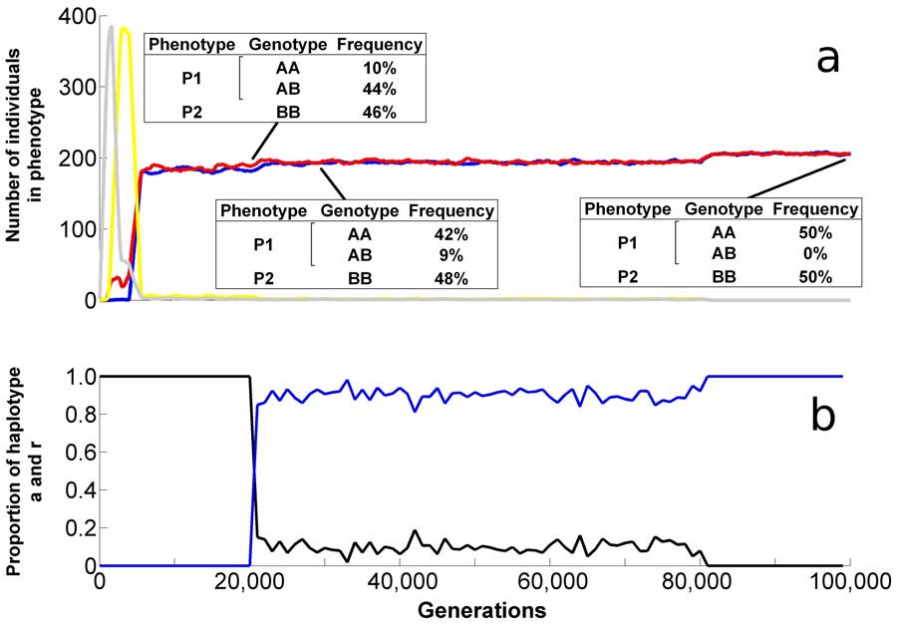
Phenotype abundances with regard to resource preference and mating preference. Trajectories in pane **a** are running averages (over 80 generations, shown every 20th generation) of phenotypes which exceeded 80 individuals in any generation: specialist P1 (red line), specialist P2 (blue), generalist on all resources (grey), and generalist in niches I–III (yellow). Detailed genotype information at generations 20,000, 30,000, and 99,000, is shown in the inserted tables. Pane **b** shows the frequency of mating gene haplotype *r* (random mating; black line) and *a* (assortative mating; blue line). The simulation is initiated with r fixed in the population, reversible mutations r↔a are applied from generation 20,000 to 80,000. Data is from the same simulation as underlying Fig. 4.

In the most common situation, after an often short but variable lag phase from the onset of mutations in the mating gene at generation 20,000, the assortative mating allele rapidly invades the population (Fig. 6b). The two haplotypes A and B (Fig. 4e) still code for the same genotypes in diploid form (Fig. 4f), but the heterozygote numbers are reduced due to assortative mating (Fig. 4f). This low proportion of heterozygotes are maintained as a result of mutational noise on the parental genes causing interbreeding among the homozygote lineages. The frequency of heterozygotes persists in mutation-selection equilibrium (Fig. 6a). In simulations with allele *r* dominant over *a*, this equilibrium exhibits a higher proportion of heterozygotes. Together with a less rapid invasion of *a*, these are the only discernable differences when dominance in the mating alleles is reversed (Table S1). When mutations in our simulations are arrested after 80,000 generations, selection removes the heterozygotes completely and creates perfect homozygote specialists mating assortatively (Fig. 6). The simulation thus demonstrates that two specialist phenotypes can arise in an environment with two favourable niches, first as stable polymorphism and then as two genetically distinct and reproductively isolated populations, provided mating takes place within niche-specific locations.

### Selection mechanism for assortative mating

The exploiters' fitness is determined by their accuracy in selecting viable (non-detrimental) resources and competition within the niches for these resources (eq. 2). In the polymorphism prior to when assortative mating is permitted, the proportion of heterozygotes that exhibit unfit phenotypes (i.e. exhibit preference for at least one detrimental resource) (11.4%) is slightly less than that of homozygotes (AA: 13.9%, and BB: 16.3%, Table 1). Thus we obtain the important insight that assortative mating is able to evolve for reasons other than selection against inferior heterozygotes, which is the case in other models of speciation. The total number of unfit phenotypes in the population as a whole was significantly lower after assortative mating was introduced (14.0%, vs. 11.6%, ; ML *χ* ^2^ = 5.97, *p* = 0.015, Table 1), thereby indicating selection for assortative mating. Since the mutation rate is kept constant, there appear to be only two possible explanations for this observation: (i) either assortative-mating phenotypes are more resistant than random-mating phenotypes to change in phenotypic expression from genomic point mutations or (ii) the removal rate of unfit phenotypes is higher in assortative mating than in random mating populations. To distinguish between these two possibilities, we subjected phenotypes selected from both randomly and assortatively mating populations to random mutations (see methods). The increase in the number of unfit phenotypes resulting from these mutational perturbations was the same for both populations (13.0% vs. 12.9% ; ML *χ* ^2^ = 0.01, n.s., Table 1) leading us to conclude that the evolution of assortative mating is associated with the benefits from a higher removal rate of unfit phenotypes.

**Table 1:** Comparison of genetic robustness in randomly (rand) and assortatively (asst) mating populations.

Genotype	AA			AB			BB			Total		
Mating	rand	asst		rand	asst		rand	asst		rand	asst	
**Unfit phenotypes (%)**	13.9	7.4	**	11.4	31.1	***	16.3	11.4	***	14.0	11.6	*
**Increase (%) in unfit phenotypes**	7.9	9.5	n.s.	8.2	0.0	***	16.5	17.6	n.s.	13.0	12.9	n.s.
**N**	187	913		962	206		1077	1127		2226	2246	

Statistics are maximum likelihood χ2 values: n.s. = not significant,

*p<0.05,

**p<0.01,

***p<0.001.

Pairs of columns show the proportion of unfit phenotypes (those responding to one or more detrimental resources) for the labeled genotypes (AA, AB, and BB), as well as the population as a whole (Total). The first row of results pertains to the proportion of unfit phenotypes just before (rand) or soon after (asst) the invasion of the assortative mating gene (Methods). The second row of results pertains to the percentage increase in unfit phenotypes after the genotypes have been subject to single point mutations (Methods). Statistics indicate significant differences in the proportion of the functional phenotypes before and after the invasion of assortative mating, indicated for each row of results.

In a population of two assortatively mating homozygote specialists AA and BB, mutations in the functional SGs are immediately expressed and subject to selection. It is due to the lower selection rate against harmful mutations that the population when mating randomly expresses a higher number of unfit phenotypes compared with the population when mating assortatively. Mutations in the silent SG (*mm*) of genotype AA will accumulate mutations over many generations as long as these genotypes keep mating assortatively. A post-zygotic reproductive barrier thus builds up, and incidental hybridization will more likely result in inferior offspring.

## Discussion

The prevailing view of sympatric speciation is that it acts through disruptive selection on adaptive quantitative traits (e.g. morphology of feeding apparatuses or on physiological systems related to detoxification of plant defensive compounds). Here we propose a complementary view in which selection acts against mutations accumulating on epistatically-acting niche-preference alleles that are only conditionally expressed in a random mating population. The two views are different with regard to adaptability, the genetic mechanisms involved, and how the selection process acts. In the prevailing view, under adaptive trait selection individuals are morphologically or physiologically canalized to perform better in one niche than the other [17], whereas in our model no such canalization exists: the niche arises purely through preference imposed by the individual's perceptual system. From a functional perspective alone, many species appear to be far more specialized than they need to be [42]. This observations suggests that niche width itself is under selection [37] probably driven by frequency-dependent emergent competition for resources (i.e. emergent in the sense of acting on the population level in contrast to the individual level). If niche preference is also a trait under disruptive selection in sympatric speciation then one would expect it to be the only trait differentiated in newly formed species. In this light, the sister species in which host choice is the only character correlated with genetic differentiation is an intriguing observation [28], [43]. We examined the sensitivity of the evolution of specialists to parameter settings in a previous study [38]. Specialists readily evolves when the range of mutational perturbations are 1/3 of the selected value. Specialists still evolves but with a lower probability even at 1/50 of the selected value, if combined with a higher mutation rate. Cross-overs at rather low rates, however, promote the evolution of a uniform population of discrimating generalists [1010] rather than two specialists. We also know that speciation can evolve in an upscaled environment with six niches, although a longer evolutionary time is required (Table S1) to see this occur.

Current speciation models typically employ additive genetics [17], [44]. Mutations in additive genes cause limited change on the expressed trait (at least in the models referred to above), and hybrids express phenotypes intermediate to their parents. In contrast, when there is disruptive selection on non-additive or epistatic genes, polymorphism readily evolves [45]. Host recognition in species such as herbivorous insects is determined by the interaction of many genes coding for receptor proteins with varying specificity [46]–[49], where individuals typically respond to ratios of signal components rather than signal strength per se [49], [50] over a moderate range of concentrations. Mutational changes can have large effects on phenotype expression with a high degree of freedom for the genotype to express phenotypes. Hybrids among genetic lineages are rarely expressing intermediate traits, as seen in tephritid flies [48]. On the contrary, they can express functional phenotypes, so that evolutionary branching can occur under random mating and establish a polymorphism (Fig. 6).

The selection mechanism in our model, ultimately acting against accumulating mutations, is different from existing theories that demonstrate how reinforcement-like selection on inferior heterozygotes selects for assortative mating [16], [17]. In both these and our models, genes for assortative mating must evolve, and as such they must also be linked to ecological or niche preference genes. A gene for mating within a specific niche, as we have included, does however, not need to be physically linked to resource preference genes if these are under disruptive selection [20]. It should also be noted that the initial polymorphism (and the following speciation) would not have evolved in case the environment had been arranged in a way that enables our ANNs to categorize the two viable niches as one superniche [37] (i.e. the opportunity exists for two kinds specialist versus one kind of generalist to emerge in our two viable niche environment). In some cases in real systems the discrimination task is trivial, e.g. to distinguish the odour of two potential host-plants that signal using compounds that do not overlap with respect to the input sensory channels (if they do overlap then ratios of compounds become important, thereby posing a greater challenge to discrimination: e.g. see [51]). Some resources, like prey, may mimic their environment being cryptic, or mimicking a noxious prey being involved in an arms race with their exploiter driven by the exploiters perceptual system [52], [53]. Evolution of asexual haploids in an environment with varying non-zero resource values, resulted in a guild with non-discriminating generalists and specialist whose population numbers matched the resource values [37]. It remains to be investigated whether or not this latter result holds for sexually reproducing organisms.

**Figure 7:**
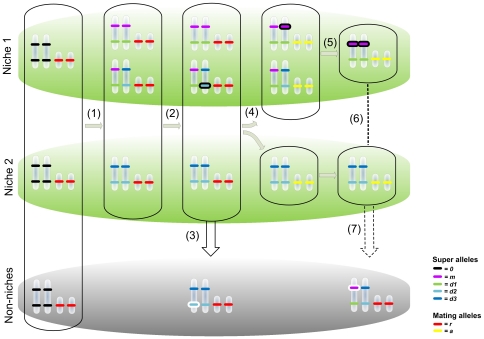
Schematic overview of the sympatric speciation process driven by selection against accumulating deleterious mutations. See *Discussion* for details.

In conclusion, our model demonstrates a new feasible process for sympatric speciation in diploid sexual organisms (Fig. 7). A non-discriminating, random mating, ancestral population appears in an environment of two niches (niche 1 and niche 2 denoted by green areas in Fig. 7) that requires sensory recognition by the exploiters. Alternatively the ancestral population initially inhabits one niche and a novel niche appears in the environment. Failure to recognize any of the two niches is detrimental to the phenotype (grey area Fig. 7). This selects for two phenotypes specialized on each resource, acting on the diploid genome with epistatic regulation of the sensory trait (1 in Fig. 7). The result is a polymorphism in which genotypes AA, and AB utilize niche 1 and genotype BB utilize niche 2, but other genotype-phenotype matching cannot be ruled out as likely until a large number of simulations have been undertaken (it will still be a challenge to calculate the likelihood of the different outcomes). Four alleles evolve in two loci (*m*, *d1*, *d2* and *d3*), which are silenced when paired *mm* in homozygote AA or *d1d2* in the heterozygote (see Fig. 5). This kind of allelic interaction readily evolves when mapping three genotypes onto two niches, most likely aided by the assumed codominance of homologous alleles. It is noteworthy that SG3 has not evolved any functional alleles at all, which may suggest that allelic interaction is not due to a size-constraint of the ANN and the genome. Silenced alleles accumulate harmful mutations (an example is marked with a black oval around the allele) in two or more generations (2 in Fig. 7). Disrupted alleles are eventually expressed in the offspring, which becomes inferior with regard to utilize an available niche (3 in Fig. 7; see Fig. 5 for a full crossing scheme). In this scenario, there is selection for assortative mating since it will reduce the proportion of unfit offspring. An allele for mating in association with the niche (*a*) is introduced to the population (4 in Fig. 7), which will spread in the population. In an assortatively mating population, the heterozygotes will be reduced by 50% by outcrossing in each generation, resulting in two genetic populations of pre-zygotically isolated (by assortatively mating) homozygote specialists. A Bateson-Dobzhansky-Muller incompatibility [25], [26] builds up by the undisturbed accumulation of mutations in genotype AA (5 in Fig. 7; marked by black encircling). Should an incidental hybridization occur (6 in Fig. 7), the hybrid is a heterozygote, more certain to have severely reduced vigor (7 in Fig. 7).

## Supporting Information

Table S1 **The table shows results from simulations with alternate settings.** Resources are depicted by a vector with resource values. Simulations had either 4 or 6 resources, of which 2 or 3 were suitable (value = 250). Resources were always lined up on the diagonal in the 2D signal space (Fig. 1). When we made shifts, resource number 2 was shifted in the signal space towards resource number 3 to limit the gene expression range in which discrimination is enabled. Network traits varied were the number of hidden nodes (3 or 4), and the dominance of the assortative mating allele in relation to the random mating allele. Other parameter settings were as described in the methods. Evolved phenotypes in these settings were assortatively mating homozygote specialists (HS), resource matching genetic polymorphism (MGP), non-matching genetic polymorphism (NGP), and discriminating generalist (DG). Multiple lineages of assortatively mating homozygote specialists evolve under all parameter settings except the last one. Recessivity/dominance of the assortative mating allele had no discernible effect on the phenotypes evolved. Larger resource vectors imply a more complex discriminating task and create more non-matching guilds at the end of simulations. Selection is expected to create resource matching solutions should the simulations have been run longer.
(DOC)
