# Supplementary material for: Selection against Accumulating Mutations in Niche-Preference Genes Can Drive Speciation
Source: PLoS One. 2011 Dec 27;6(12):e29487. doi: 10.1371/journal.pone.0029487 (PMC3246506; doi:10.1371/journal.pone.0029487)
Supplement: Table S1 — The table shows results from simulations with alternate settings. Resources are depicted by a vector with resource values. Simulations had either 4 or 6 resources, of which 2 or 3 were suitable (value = 250). Resources were always lined up on the diagonal in the 2D signal space (Fig. 1). When we made shifts, resource number 2 was shifted in the signal space towards resource number 3 to limit the gene expression range in which discrimination is enabled. Network traits varied were the number of hidden nodes (3 or 4), and the dominance of the assortative mating allele in relation to the random mating allele. Other parameter settings were as described in the methods. Evolved phenotypes in these settings were assortatively mating homozygote specialists (HS), resource matching genetic polymorphism (MGP), non-matching genetic polymorphism (NGP), and discriminating generalist (DG). Multiple lineages of assortatively mating homozygote specialists evolve under all parameter settings except the last one. Recessivity/dominance of the assortative mating allele had no discernible effect on the phenotypes evolved. Larger resource vectors imply a more complex discriminating task and create more non-matching guilds at the end of simulations. Selection is expected to create resource matching solutions should the simulations have been run longer. (DOC) [file pone.0029487.s001.doc]

**Table S1.** Summary of simulation results.

| **Resources** | | **Network** | | **Evolved phenotypes** | | | |
| --- | --- | --- | --- | --- | --- | --- | --- |
| **Vector** | **Shift** | **Nodes** | **Assortative mating** | **HS** | **MGP** | **NGP** | **DG** |
| [250, 0.01, 250, 0.01] | medium | 3 | Recessive | 8 | 2 |  |  |
| [250, 0.01, 250, 0.01] | medium | 3 | Dominant | 8 | 2 |  |  |
| [250, 0.01, 250, 0.01] | small | 3 | Recessive | 9 |  |  | 1 |
| [250, 0.01, 250, 0.01] | small | 3 | Dominant | 10 |  |  |  |
| [250, 0.01, 250, 0.01] | large | 3 | Recessive | 9 | 1 |  |  |
| [250, 0.01, 250, 0.01] | large | 3 | Dominant | 9 | 1 |  |  |
| [0.01, 0.01, 250, 0.01, 250, 0.01] | none | 3 | Dominant | 2 |  | 3 |  |
| [0.01, 0.01, 250, 0.01, 250, 0.01] | none | 4 | Dominant | 4 | 1 |  |  |
| [250, 0.01, 250, 0.01, 250, 0.01] | none | 3 | Dominant | 3 |  | 2 |  |
| [250, 0.01, 250, 0.01, 250, 0.01] | none | 4 | Dominant |  |  | 5 |  |
